# IPNs from Cyclodextrin:Chitosan Antioxidants: Bonding, Bio-Adhesion, Antioxidant Capacity and Drug Release

**DOI:** 10.3390/jfb5030183

**Published:** 2014-09-17

**Authors:** V. Tamara Perchyonok, Sias R. Grobler, Shengmiao Zhang

**Affiliations:** 1 VTPCHEM PTY Ltd, Glenhuntly, Melbourne 3163, Australia; 2 Oral and Dental Research Institute, Faculty of Dentistry, University of the Western Cape, Private Bag X1, Tygerberg 7505, Cape Town, South Africa; E-Mail: siasgrobler1@gmail.com; 3 School of Material Science and Engineering, East China University of Science and Technology, 130 Meilong Road, Shanghai 200237, China; E-Mail: shmzhang@ecust.edu.cn

**Keywords:** IPN, chitosan, β-cyclodextrin, antioxidants, cumulative release, free radical damage

## Abstract

IPNs are unique “alloys” of cross-linked polymers in which at least one network is synthesized and/or cross-linked in the presence of the other. IPNs are also known as entanglements of polymer networks that are ideally held together only by permanent topological interactions. The objectives of this study are to evaluate novel chitosan-based functional drug delivery systems that can be successfully incorporated into “dual action bioactive tooth restorative materials”. These materials should be capable of inducing an improved wound healing prototype. The novel hydrogels will be investigated with respect to the antioxidant capacity of conventional antioxidants, such as resveratrol, β-carotene and propolis, as a designer drug delivery system, with the use of SEM imaging for the characterization of the surfaces, bio-adhesive property, antioxidant capacity, free radical defence, antioxidant, active ingredient stability and reactive features of novel materials. The additional benefit of the site-specific “functional restorative material” for use in dressings to deliver antibiotics to wound sites can provide tissue compatibility and reduced interference with wound healing. The materials were tested using an effective* in vitro* free radical generation model as functional additive prototypes for further development of “dual function restorative wound healing materials”. We quantified the effects of functional designer biomaterials on the dentin bond strength of a composite and evaluated the bio-adhesive capacity of the materials in the two separate “*in vitro*” systems. The added benefits of the chitosan/vitamin C/cyclodextrin (CD) host:guest complex-treated hydrogels involved a positive influence on the tetracycline release, increased dentin bond strength, as well as a demonstrated* in vitro* “built-in” free radical defence mechanism and, therefore, acting as a “proof of concept” for functional multi-dimensional restorative wound healing materials with a built-in free radical defence mechanism. Based on our results, we can conclude that the CD:chitosan-antioxidant-containing hydrogels are a suitable carrier for tetracycline to be slow-released. Within the limitations of the study design, chitosan-based hydrogels are suitable materials for functional restorative and wound healing applications* in vitro*. Cytotoxicity data are currently being evaluated in our laboratory.

## 1. Introduction

IPNs are unique “alloys” of cross-linked polymers in which at least one network is synthesized and/or cross-linked in the presence of the other. IPNs are also known as entanglements of polymer networks that are ideally held together only by permanent topological interactions [1]. The inter-network entanglements are permanent because of chemical cross-linking and cannot be separated. Many researchers have suggested that IPN formation enables the enhancement of the performance of hydrogels. Generally, IPNs are created for the purpose of combining individual properties of two or more polymers. In some cases, entirely new properties are exhibited by the IPN that are not observed in either of the two single networks alone [2]. The development of interpenetrating network polymers is attractive, because IPNs provide free volume space for the easy encapsulation of drugs in the three-dimensional network structure, which is obtained by the cross-linking of two or more polymer networks [3]. Various properties of IPNs, such as porosity, bio-adhesiveness, elasticity, swelling and stimuli-responsive behaviour, can be controlled by the appropriate choice of the network-forming polymers and suitable cross-linking agent and its proportion [4,5].

Bio-adhesive polymers appear to be particularly attractive for the development of an alternative etch-free dentin bonding system with the added advantage of additional therapeutic delivery systems to improve intra-dental administration of therapeutic and prophylactic agents if necessary [6,7]. Chitosan and cyclodextrins, which are biologically safe biopolymers, have been proposed as bio-adhesive polymers and are of continuous interest to us, due to their unique properties and flexibility in a broad range of oral applications reported by others and us recently [8,9].

Chitosan is a linear poly-saccharide composed of randomly distributed β-(1,4)-linked D-glucosamine and *N*-acetyl-D-glucosamine units. Chitosan is structurally similar to glycosaminoglycans and has excellent biocompatibility, low toxicity and immune-stimulatory activities [10]. Chitosan can be used in a minimally-invasive manner, because it can undergo thermal and pH-triggered gelation and be enzymatically degraded* in vivo* by lysozyme and chitosanase enzymes [11]. Injectable chitosan-based gels have been employed for drug delivery [12] and tissue engineering of bone [13], cartilage [14], nerve [15], human intervertebral disc (IVD) [16], as well as for brain cancer treatment [17].

Cyclodextrins (CD) are cyclic oligosaccharides capable of forming non-covalent inclusion complexes with a variety of drugs, including proteins [18]. CD complex formation often improves the physicochemical and biological properties of guest molecules [19]. CD complexes with protein drugs, such as insulin, might drastically reduce their state of aggregation in solution [20]. The hydrophobic domains in the protein molecule can penetrate into the non-polar CD cavity, leading to the formation of non-covalent inclusion complexes [21]. Apart from the complexation aspects, hydrophobic derivatives of CD, such as MCD, are also known to enhance the absorption of hydrophilic molecules across biological barriers [22,23].

Combining CD complexed systems with polymeric carriers seems to be an interesting strategy in improving oral drug delivery [24]. CD complexes may help in enhancing drug stability/absorption, while the particulate delivery system may serve as a platform for the encapsulation of the complexed drugs [25].

The objectives of this study are to evaluate novel chitosan-based functional drug delivery systems that can be successfully incorporated into “dual action bioactive tooth restorative materials”. These materials should be capable of inducing an improved wound healing prototype and containing common antioxidants/therapeutic agents (propolis, resveratrol and β-carotene) to promote healing.

The novel hydrogels will be investigated with respect to the antioxidant capacity of conventional antioxidants, such as resveratrol, b-carotene and propolis, as a designer drug delivery system, with the use of SEM imaging for the characterization of the surfaces, bioadhesive property, antioxidant capacity, free radical defence, antioxidant, active ingredient stability and reactive features of novel materials. The additional benefit of the site-specific “functional restorative material” for use in dressings to deliver antibiotics to wound sites can provide tissue compatibility and reduced interference with wound healing.

## 2. Results and Discussion

### 2.1. The Characterization of Prepared Gels (Gel 1–Gel 4)

**Figure 1:**
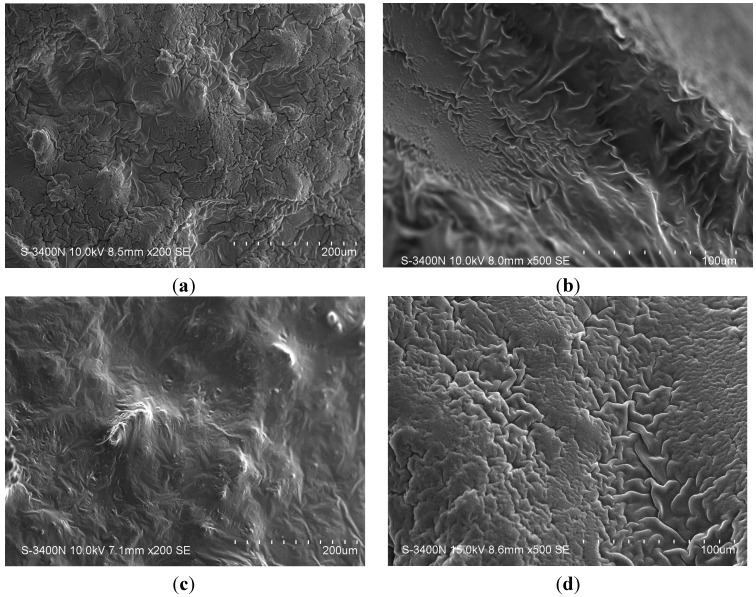
SEM photographs of the interior morphology of the selected gels under investigation: (**a**) Gel 1; (**b**) Gel 2; (**c**) Gel 3 and (**d**) Gel 4.

SEM images were obtained to characterize the microstructure of the freeze-dried gels and are presented in Figure 1. It could be seen that the gels displayed a homogeneous pore structure similar to a sponge. SEM analysis revealed interconnected pores of different sizes and flat, relatively smooth walls. The biomaterial remained intact after 24 days of immersion in artificial saliva, as was confirmed by SEM. It was thought that the micro-porous structure of the gels could lead to high internal surface areas with low diffusional resistance in the gels. The surfaces of the gels were also presented (Figure 1). The “skin” of the gels can be seen, and the collapse of the surface pores may be due to artefacts (freeze-drying process).

### 2.2. Studies of Equilibrium Swelling in Chitosan Gels (Gel 1–4)

**Figure 2:**
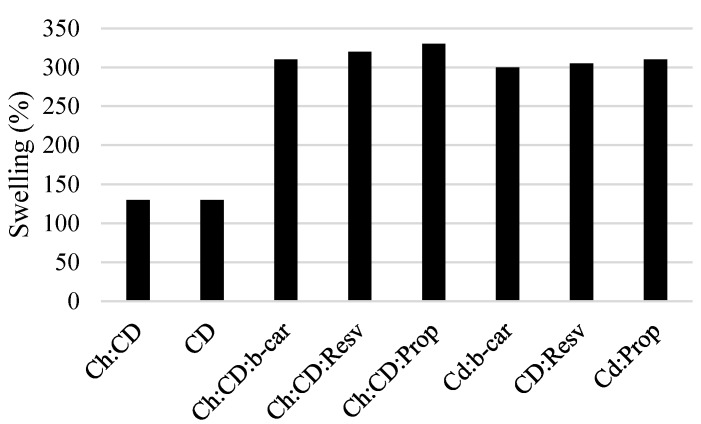
The degree of water uptake of the gels, Gel 1–Gel 4, and β-cyclodextrin inclusion complexes for comparison (*n* = 3, *p* < 0.05). CD, cyclodextrin; Ch, chitosan; Prop, propolis; b-car, β-carotene; Resv, resveratrol.

The hydrogels remain in the cylindrical form after swelling. Compared with dry state hydrogels, the volume of the swollen state hydrogel displays a significant increase, as summarized in Figure 2.

### 2.3. Bond Strength Testing

**Figure 3:**
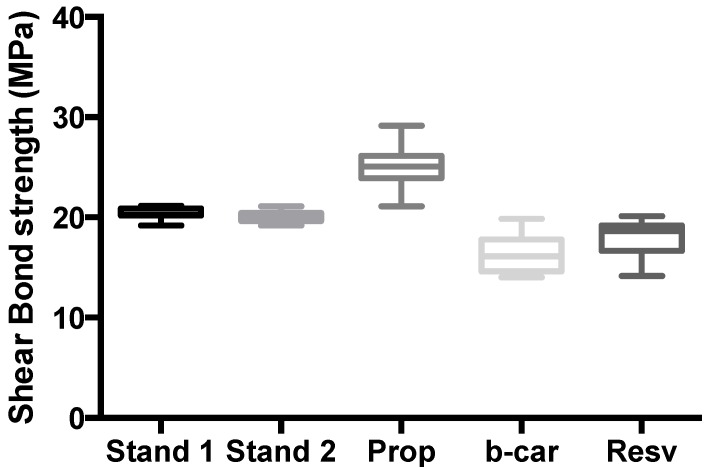
Shear bond strength of hydrogels after 24 h of bonding to dentin. The maximum and minimum values were given. The intermediary box represents the position of 50% of the values, and the line within the box shows the median values.

**Figure 4:**
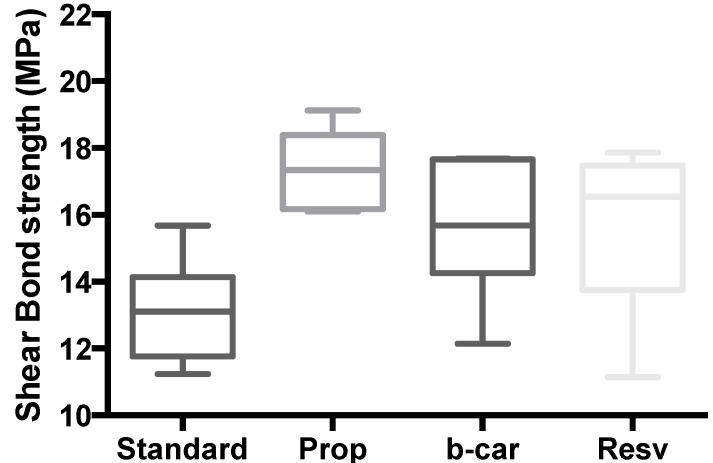
Shear bond strength of hydrogels after three months of bonding to dentin. The maximum and minimum values are given. The intermediary box represents the position of 50% of the values, and the line within the box shows the median values.

The shear bond strength values (MPa) of the composite restorations were given after 24 h (Figure 3), as well as after three months (Figure 4).

Mean shear bond strength values and the difference between the groups are summarized in Figure 3 for bonding to dentin after 24 h and, in Figure 4, after three month. In general, there was an increase in bond strength of the dentin treated with the CD:chitosan:Vit C (vitamin C) complex-containing hydrogels compared to the bond strength of the conventionally-bonded teeth. Interestingly, the increase in bond strength was also observed in groups of hydrogen peroxide-exposed samples [26], suggesting that there are additional benefits associated with the chitosan:therapeutic-agent:antioxidant system that are in need of further investigation.

The results of this study suggest that optimum results for the strengthening of dentin can be achieved throughout the immediate treatment with the antioxidant:chitosan:vitamin C “host:guest” complex with the increase of dentin bond strength. This additional advantage of the system may suggest that antioxidant release from chitosan gel depends on the physical host:guest structure, as well as the pH properties and flexibilities of the material.

The additional benefit of using the chitosan:antioxidant system as a bonding/pre-bonding to enamel and dentin system lies in its ability to show favourable immediate results in terms of bonding effectiveness, as well as the durability of resin-dentin bonds for a prolonged time (up to 6 months) [26]. It is well documented that the hydrostatic pulpal pressure, the dentinal fluid flow and the increased dentinal wetness in vital dentin can affect the intimate interaction of certain enamel and dentin adhesives with dentinal tissue [27]. Therefore, the newly-developed chitosan-derivatised systems support our earlier reported results, being able to address the shortfalls affecting the long-term bonding performance of modern adhesives and addressing the current perspectives for improving the bond durability of conventional adhesive systems, as demonstrated in our “*in vitro*” model of restoration repair and corresponding shear bond strength, especially in the β-CD/chitosan/propolis host:guest system [27].

### 2.4. Free Radical Defence Capability of the Prepared Hydrogels

When wounds occur, this is generally accompanied by classical symptoms of inflammation, such as pain, redness and oedema. The inflammation stage begins immediately after injury: first vasoconstriction, platelet aggregation at the injury site and then infiltration of leukocytes and the T lymphocytes to the wound area. The cicastration process proceeds naturally, since the damaged tissue attempts to re-establish haemostasis. The amount of uncontrolled ROS is the main cause of the inability of the healing process to continue, and therefore, it would be ideal to utilize the antioxidant capacity of the “designer hydrogels” to detect and be able to “fight the free radical excess”. This has been assessed using a previously described model, where the HO radical can be generated from a reaction known as the biologic Fenton reaction, and this reaction requires the presence of H_2_O_2_ [28]. Bovine serum albumin (BSA), a completely water-soluble protein, was polymerized by hydroxyl radicals generated by the Fenton reaction system of Fe^2+^/EDTA/H_2_O_2_/ascorbate [28]. As a result, the protein loses its water-solubility and the polymerized product precipitates. The decrease in the concentration of the water-soluble protein can easily be detected.

**Figure 5:**
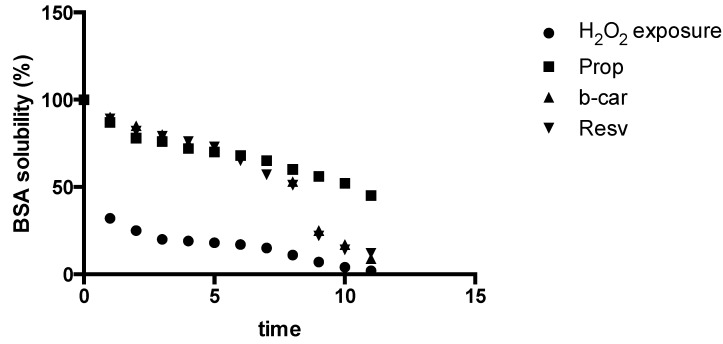
Plot of changes in water solubility of the model protein bovine serum albumin (BSA) over time (12 h) exposed to free radicals generated by a Fe^2+^/EDTA/H_2_O_2_/ascorbate system as a source of free radicals.

We considered it worthwhile to study the chitosan/β-cyclodextrin as a “built-in defence mechanism” for the* in vitro*-generated free radical production and “site-specific”* in vitro* model counter reaction of the hydrogel. Therefore, we adopted a method for recording changes in the water solubility of the model protein, bovine serum albumin (BSA), exposed to free radicals generated by an inorganic chemical system. As clearly demonstrated by Figure 5, upon exposure to standard H_2_O_2_ in the form of Fe^2+^/EDTA/H_2_O_2_/ascorbate solution as a baseline determinate for free radical generation under “prototypical* in vitro* free radical damage”, upon incorporation of the chitosan substituted hydrogels, the built-in antioxidant capacity and, therefore, free radical defence of the* in vitro* model have been activated and are of a significant value for which notice should be taken. This model represents the practical approach of *in situ* monitoring and testing of the amount of the free radical production and synergistic antioxidant defence of the system. Further investigations with fine-tuning of the system are currently on the way in our laboratory.

### 2.5. Bio-Adhesion in Vitro Model

The higher adhesiveness of the gels is desired to maintain intimate contact with skin or the tooth structure, and the results are summarized in Table 1. Chitosan hydrogels showed the highest adhesive force and work of adhesion, which can be expected, because of the well-known intrinsic bioadhesive properties of chitosan [26,27,28,29,30]. The adequate water absorption capacity together with the cationic nature, which promotes binding to the negative surface of the skin or dentin structure, can also interpret this results.

**Table 1:** *In vitro* bio-adhesion test.

Hydrogel	Adhesive Force (N) ± SD (Skin)	Adhesive Force (N) ± SD (Dentin)	Work of Adhesion (N·cm) ± SD (Skin)	Work of Adhesion (N·cm) ± SD (Dentin)
Gel 1	1.450 ± 0.30	1.71 ± 0.35	4.35 ± 0.48	5.92 ± 0.34
Gel 2	1.02 ± 0.27	1.17 ± 0.44	2.19 ± 0.52	2.49 ± 0.42
Gel 3	1.01 ± 0.30	1.12 ± 0.60	2.85 ± 0.41	2.94 ± 0.29
Gel 4	1.67 ± 0.30	1.81 ± 0.35	5.15 ± 0.48	6.12 ± 0.34

### 2.6. The Presented Values Are an Average of 5 (n = 5)

Chitosan/Vit/Cd (Gel 1) and Chitosan/Vit/CD/propolis (Gel 4) showed the highest adhesive force and work of adhesion. This can be expected, because of the well-known intrinsic bio-adhesive properties of chitosan and propolis. The adequate water absorption capacity together with the cationic nature, which promotes binding to the negative surface of skin and the dentin structure, can also be used to interpret these results. According to Caffaggi, hydration of the polymer causes mobilization of the polymer chains and, hence, influences polymeric adhesion [30]. Appropriate swelling is important to guarantee adhesivity; however, over-hydration can form slippery non-adhesive hydrogels [30]. In addition, for the molecular arrangement of the polymeric chains, which are present in the new hydrogels, such as with propolis, the correlation between the force and work of adhesion is noticeable for all. Further experiments are to be conducted on skin samples to further evaluate the bio-adhesive capacity of the designer hydrogels.

## 3. Discussion

### Drug Release Studies in Vitro, Propolis, β-Carotene and Resveratrol as Poorly Water Soluble Prototypes in Chitosan/Vit/CD/Propolis Hydrogel

**Figure 6:**
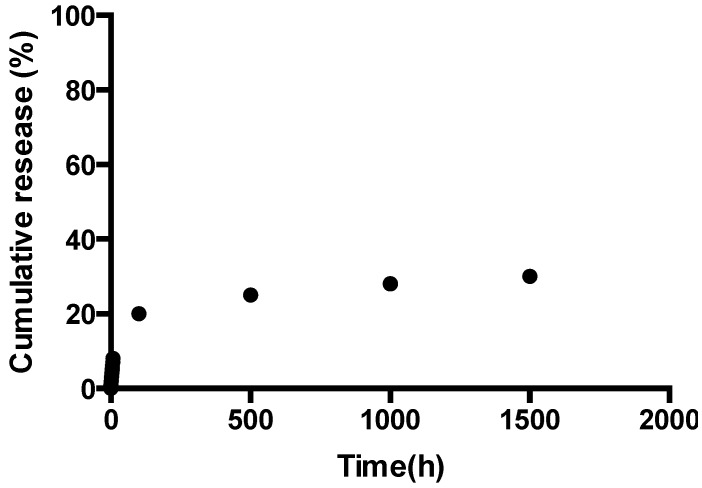
Slow-release profile of Gel 4 as a function of time with propolis as the active ingredient.

In the slow-release test, the components of propolis were soluble in artificial saliva, as we could quantify total flavonoids in this medium. As it does simulate the oral environment, this test was potentially indicative of the release of propolis and are summarized in Figure 6. 

The release of Gel 4, containing ethanolic propolis extract as a potential therapeutic agent prototype, remained stable in the early hours of the experiment, allowing a more constant release, which would ensure effective and prolonged antimicrobial activity when applied clinically. These relevant characteristics for the control of cariogenic biofilm make the system a suitable candidate for further development as a functional dual action restorative material.

**Figure 7:**
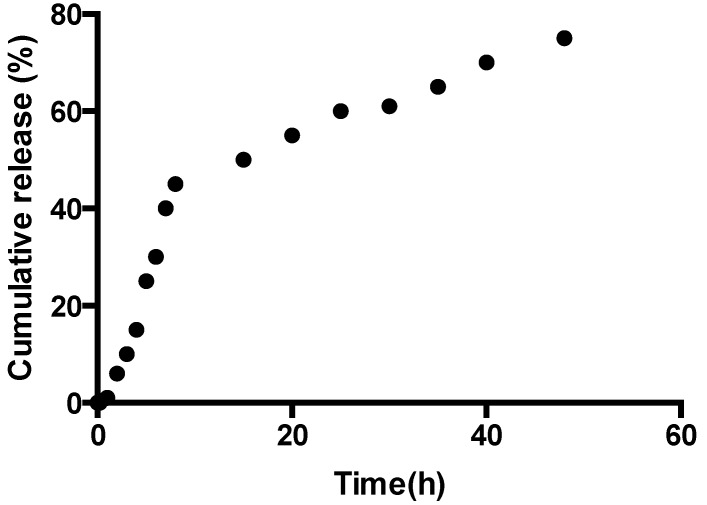
Release profile of Gel 3 as a function of time with resveratrol as the active ingredient.

The resveratrol release from Gel 3 (chitosan/Vit/CD/resveratrol) can be summarized in Figure 7. The controlled release of resveratrol depends on the pH value of the media, with slower release kinetics at the higher pH conditions, which was attributed to the amino groups of chitosan protonation, resulting in a soluble and positively-charged polysaccharide, leading to faster swelling in the acidic media. The release data are represented as the fractional release data and follow the Higuchi model. The plot reveals that there are two stages for the release of the resveratrol from Gel 3. The first stage of release was initially rapid (burst release), which may be the result of the rapid diffusion of resveratrol from the chitosan/Vit/CD/resveratrol hydrogel and the initial swelling. The second stage of release of the resveratrol demonstrates a slow, controlled release. The burst release should have the advantage of reaching a biologically relevant concentration much faster, whereas the following slow release (Figure 7) will control the sustainable concentration of resveratrol in plasma, for example for a prolonged period of time. This outcome will suggest the poor bioavailability of resveratrol, because of the rapid metabolism and elimination can benefit from the chitosan/Vit/CD/resveratrol encapsulation.

**Figure 8:**
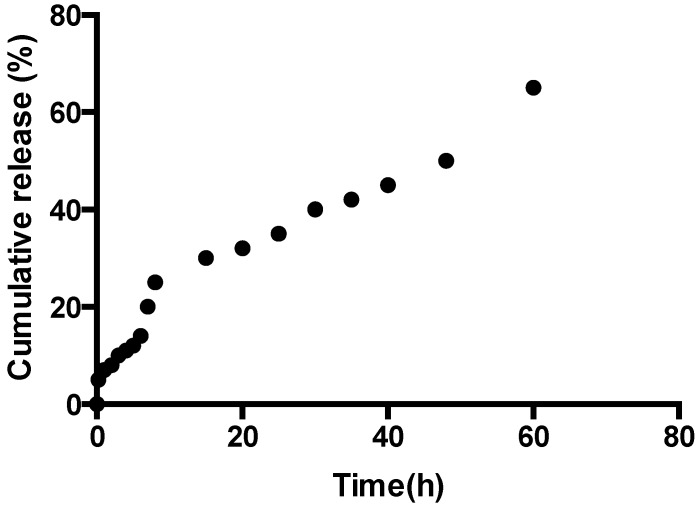
Release profile of Gel 2 as a function of time with β-carotene as the active ingredient.

In the case of β-carotene, we have found that the release pattern is as previously observed and is correlated with the swelling ability of the corresponding hydrogel. Figure 8 shows the relationship between the release time and the percentage of β-carotene released. The release of beta-carotene is gradual and finally reaches the equilibrium state, where the β-carotene is no longer released. The advantage of the chitosan/Vit/CD/β-carotene system is the extension of the time of the release of β-carotene, as well as the protection of the active ingredient from any type of free radical damage produced in and around the active site.

## 4. Experimental Section

Chitosan:vitamin C (5:1) (Aldrich, Sydney, Australia), β-cyclodextrin (Aldrich), glycerol (Sigma, Sydney, Australia) and glacial acetic acid (E. Merck, Sydney, Australia) were used as received. The degree of de-acetylation of typical commercial chitosan used in this study is 87%. The isoelectric point is 4.0–5.0. Resveratrol, β-carotene and propolis (Aurora Pharmaceuticals, Sydney, Australia) were used as bought.

### 4.1. Methods

The hydrogels were prepared as previously reported in our protocol [26]. The physico-chemical features included surface morphology (SEM), release behaviours, stability of the therapeutic agent-antioxidant-chitosan and the effect of the hydrogels on the shear bond strength of dentin, which were measured and compared to the earlier reported IPN β-CD/chitosan-antioxidant-containing hydrogels. Bio-adhesive studies were performed in order to assess the suitability of these designer materials. The free radical defence capacity of the biomaterials was also evaluated.

### 4.2. Preparation of Hydrogels Containing Various Antibiotics

Chitosan:β-CD hydrogels have been prepared using the previously described methodology [26]. Briefly, the corresponding antibiotic and antioxidant mixtures were incorporated by dispersion of the corresponding antioxidant powder, 0.2 grams, in glycerol (5% w/w) using a mortar and a pestle and 1 mL of glacial acetic acid (2% w/w). The corresponding antioxidant mixtures were incorporated into the mixture, and the summary of the newly prepared materials is specified in Table 2.

**Table 2:** Gel formulation prepared in the study. CD, cyclodextrin; Vit C, vitamin C; Ch, chitosan.

Gel Formulation	Gel Number	Chitosan /Vitamin C (5:1) (w/w%)	Antioxidant	β-Cyclodextrin (w/w%)	pH
Ch/Vit C/CD	Gel 1	5	0	5	6.12
Ch/Vit C/CD/β-carotene	Gel 2	5	1	5	5.98
Ch/Vit C/CD/resveratrol	Gel 3	5	1	5	6.24
Ch/Vit C/CD/propolis	Gel 4	5	1	5	6.13

### 4.3. Determination of Gel pH

One gram of the prepared gels was accurately weighed and dispersed in 10 mL of purified water. The pH of the dispersions was measured using a pH meter (HANNA instruments, HI8417) [26].

Infusion bags containing a known weight of dry gels were immersed in pH 4.0 and pH 9.0 buffer solutions, respectively, and kept at 25 °C for 48 h until equilibrium of swelling had been reached. The swollen gels were taken out and immediately weighed with a microbalance after the excess of water lying on the surfaces was absorbed with a filter paper. The equilibrium swelling ratio (SR) was calculated using the following equation:

SR = (*W*_s_ − *W*_d_)/*W*_d_ × 100%

where *W*_s_ and *W*_d_ are the weights of the gels at the equilibrium swelling state and at the dry state, respectively [21,22,23]. Experiments were repeated in triplicate for each gel specimen and the mean value calculated.

### 4.4. Bio-Adhesive Investigation

Bio-adhesion studies were done using a Chatillon apparatus for force measurement [27]. This method determines the maximum force and work needed to separate two surfaces in intimate contact [27]. The hydrogels (0.1 g) were homogeneously spread on a 1-cm^2^ glass disk, and then, the disks were fixed to the support of the tensile strength tester using double-sided adhesive tape. The gel was brought into contact with a commercially available band aid, in order to simulate the skin attachment, or contact with a slice of dentin was established in order to imitate the adhesion of the gel to the tooth structure. After a pre-set contact time (1 min) under contact strength (0.5 N), the 2 surfaces were separated at a constant rate of displacement (1 mm/s). The strength was recorded as a function of the displacement, which allowed us to determine the maximal detachment force (*F*_max_) and the work of adhesion (*W*), which was calculated from the area under the strength-displacement curve.

### 4.5. Morphology of the Gels

The samples were prepared by freezing in liquid nitrogen for 10 min and then were freeze-dried for 24 h. The prepared samples were fractured in liquid nitrogen using a razor blade. The fractured samples were dried under vacuum, attached to metal stubs and sputter coated with gold under vacuum for the SEM study. The interior and the surface morphology were observed under scanning electron microscope (Hitachi S4800, Hitachi High Technologies Corporations, Tokyo, Japan).

### 4.6. Gel Stability 

The stability of the gel formulations was also investigated. The organoleptic properties (colour, odour), pH, drug content and release profiles of the gels stored at 20 °C were examined on Days 0, 15, 30 and 178. The performance of the hydrogels was not affected by the storage conditions, suggesting the remarkable stability of the novel biomaterials under investigations.

### 4.7. Shear Bond Strength Tests for Dentin Bonding

Extracted non-carious, intact, human molars stored in water containing a few crystals of thymol at 4 °C were used within two months using the protocol previously described by us [26]. 

Forty eight teeth samples were prepared and divided into 6 groups of 8 each, A–F (Table 3), and stored in a solution of artificial saliva. These groups were then treated as outlined in Table 3. After 24 h, one stud of the two of each tooth was tested for shear bond strength. An Instron Universal Testing Machine at a crosshead speed of 0.5 mm/min was used to test the de-bonding strength. All data were analysed for statistical significance using the non-parametric ANOVA test.

**Table 3:** Groups tested (8 teeth per group).

Samples	Shear Bond Strength Testing Conditions
Group A	37% of phosphoric acid + primer + bonding immediately (negative control)
Group B	Self-etching primer + bonding immediately (positive control)
Group C	Gel 1 + bonding immediately
Group D	Gel 2 + bonding immediately
Group E	Gel 3 + bonding immediately
Group F	Gel 4 + bonding immediately

## 5. Conclusions

The materials were tested using an effective* in vitro* free radical generation model as functional additive prototypes for further development of “dual function restorative wound healing materials”. We quantified the effects of functional designer biomaterials on the dentin bond strength of a composite and evaluated the bio-adhesive capacity of the materials in the two separate “*in vitro*” systems. The added benefits of the chitosan/vitamin C/CD host:guest complex-treated hydrogels involved a positive influence on the tetracycline release, increased dentin bond strength, as well as a demonstrated* in vitro* “built-in” free radical defence mechanism and, therefore, acting as a “proof of concept” for functional multi-dimensional restorative wound healing materials with a built-in free radical defence mechanism. Based on our results, we can conclude that the CD:chitosan-antioxidant-containing hydrogels are a suitable carrier for tetracycline to be slow-released. Within the limitations of the study design, chitosan-based hydrogels are suitable materials for functional restorative and wound healing applications* in vitro*. Cytotoxicity data are currently being evaluated in our laboratory.
